# 113 Physical Activity for Health Pilot: Making Physical Activity inclusive for people living with chronic conditions

**DOI:** 10.1093/eurpub/ckae114.021

**Published:** 2024-09-26

**Authors:** Louise Burke

**Affiliations:** Sport Ireland, Dublin, Ireland

## Abstract

Physical Activity for Health (PAFH) is a pilot initiative funded by Sláintecare in collaboration with Sport Ireland where 6 Local Sports Partnerships (LSPs) across the country house a PAFH Officer. Limerick, Longford, Wexford, Wicklow, Waterford and Mayo LSPs were selected for the PAFH pilot and have PAFH officers in post since July 2023.

The LSPs were established in 2001 to help people get active and remove barriers to participation in sport and physical activity. The LSPs aim to create opportunities for all members in the community to be more active with a focus on moving people from inactivity to activity and traditionally focused on people in target groups like women and girls, and people with disabilities. It is now recognised that people living with a chronic condition is a target group that can be reached through the PAFH officer.

The aim of the pilot is to improve pathways for those living with chronic conditions to engage in community based physical activity. The PAFH Officer will bridge the gap that exists for those with a chronic condition who have left the healthcare system and are unable to participate in current LSP programmes. The aim is to ensure that those who need more attention and care will be looked after within these physical activity programmes until they are ready to engage in community based physical activity.

The PAFHOs have engaged with many key stakeholders across the health service, voluntary sector and third level institutions to date and will continue to further expand on this work going forward. The 6 PAFH Officers are in post as of July 2023 and have engaged directly with the public with delivery of programmes by exercise professionals trained to work in this space.

Example of the programmes being delivered include MoveWell: Breathe Well | Limerick Sports Partnership and Walking for Chronic Conditions/Longford Sports Partnership. MoveWell: Activator Walking for Neuro Conditions | Limerick Sports Partnership

The presentation at HEPA will outline the journey to date, lessons learned and the impact of the PAFH pilot on its participants.

